# Prevalence and Factors Associated with the Reuse of Mask during the COVID-19 Pandemic: A Nationwide Survey in Taiwan

**DOI:** 10.3390/ijerph18158065

**Published:** 2021-07-29

**Authors:** Feng-Shiang Cheng, Yung-Feng Yen, Shu-Yi Lin, Shih-Han Weng, Yi-Chang Chou, Dachen Chu, Chu-Chieh Chen, Hsiao-Yun Hu

**Affiliations:** 1 Department of Education and Research, Taipei City Hospital, Taipei 106, Taiwan; A3989@tpech.gov.tw (F.-S.C.); DAM37@tpech.gov.tw (Y.-F.Y.); A3810@tpech.gov.tw (S.-Y.L.); A4391@tpech.gov.tw (S.-H.W.); T0036@tpech.gov.tw (Y.-C.C.); 2 Institute of Public Health, National Yang Ming Chiao Tung University, Taipei 112, Taiwan; 3 Section of Infectious Diseases, Taipei City Hospital, Yangming Branch, Taipei 111, Taiwan; 4 Department of Health Care Management, National Taipei University of Nursing and Health Sciences, Taipei 112, Taiwan; chuje@ntunhs.edu.tw; 5 Department of Psychology and Counseling, University of Taipei, Taipei 100, Taiwan; 6 Department of Health and Welfare, University of Taipei, Taipei 100, Taiwan; DAD57@tpech.gov.tw; 7 Department of Neurosurgery, Taipei City Hospital, Taipei 103, Taiwan; 8 Institute of Hospital and Health Care Administration, National Yang Ming Chiao Tung University, Taipei 112, Taiwan

**Keywords:** COVID-19, SARS-CoV-2, reuse of masks, knowledge on usage of masks

## Abstract

Mask usage is an effective measure to prevent severe acute respiratory syndrome coronavirus 2 (SARS-COV-2) infection; however, mask reuse is not recommended. Studies examining the factors associated with mask reuse during the coronavirus disease (COVID-19) pandemic are limited. This nationwide survey aimed to determine the prevalence and factors associated with mask reuse among Taiwanese citizens during the pandemic. From 18 May through 31 May 2020, a computer-assisted telephone interview system was used to randomly select Taiwanese citizens for interview regarding COVID-19-preventive behaviors and knowledge on mask usage. For a total of 1075 participants, the overall mean age was 57.4 years, and 82.2% of participants reported mask reuse during the COVID-19 pandemic. After controlling for other covariates, participants who had a greater knowledge of mask usage or had a high supply of masks were less likely to reuse masks during the pandemic. Moreover, generalized estimating equations (GEE) analysis showed that, compared with the participants’ mask-wearing behaviors before the COVID-19 pandemic, they were more likely to reuse masks during the pandemic. Thus, it is imperative to educate people on the correct usage of masks. Furthermore, the government should provide sufficient masks to the general population to reduce mask reuse.

## 1. Introduction

Coronavirus disease (COVID-19) is caused by severe acute respiratory syndrome coronavirus 2 (SARS-CoV-2), and it was first detected in Wuhan, China, in December 2019. COVID-19 transmission has rapidly accelerated, resulting in it becoming a pandemic within a short period [1], and as of 12 July 2021, more than 186.4 million individuals have been infected with COVID-19 globally, with the official death toll reaching 4.0 million [2].

### 1.1. COVID-19 Prevention

As the number of COVID-19 patients continues to increase, data increasingly show that not only do medical staff in hospitals require face masks as personal protective equipment [3], but the effective reproduction number (R0) can also be decreased below 1 when face masks are continuously used by the general public, mitigating the spread of the pandemic [4]. To prevent the COVID-19 outbreak, Taiwan Centers for Disease Control (CDC) has implemented several strategies, including public use of masks, border control, and quarantine procedures since early January 2020 [5,6,7]. By 12 July 2021, 15,273 laboratory-confirmed COVID-19 cases were reported by Taiwan CDC, including 1284 (8.4%) in those entering Taiwan [8]. The national mortality rate in laboratory-confirmed COVID-19 cases was 4.9% [8].

COVID-19-prevention measures include pharmacological and nonpharmacological interventions [9,10,11]. Of all pharmacological interventions, vaccination is the most effective strategy to prevent SARS-CoV-2 infection. Two recent reports showed that the Pfizer-BioNTech or Moderna two-dose COVID-19 vaccine regimen had a 94–95% efficacy at preventing symptomatic SARS-CoV-2 infection [10,11]. Although vaccine is the best tool to control the COVID-19 pandemic, vaccine shortages occur in many countries [12,13,14] in 2021. Therefore, nonpharmacological public health measures, such as wearing a face mask, quarantine, isolation, social distancing, and community containment, are still important and effective ways to prevent and control the COVID-19 outbreak before adequate vaccine availability [9].

### 1.2. About Reusing Disinfection Masks

In most countries, wearing masks in public has been an essential health protection measure during the COVID-19 pandemic. Wearing a face mask is the mainstay non-pharmaceutical intervention (NPI) to prevent being infected by SARS-CoV-2 [15]. This NPI could be effectively implemented at minimum cost and without remarkably disrupting social practices. Although the guidelines for wearing a face mask vary considerably across countries [16], there is a consensus that symptomatic individuals and those in healthcare settings should use face masks [17]. However, re-use of disposable masks is not recommended because of the potential risk of wearing a SARS-CoV-2-contaminated mask. Chin et al. reported that SARS-CoV-2 can survive on masks for as long as seven days [18].

There are currently some strategies to protect people from reusing medical masks, such as dry heat, ultraviolet germicidal irradiation, moist heat incubation, and hydrogen peroxide vapor. Most of these reports focused on the disinfection of masks in medical institutions [19,20,21,22]. However, reuse of disposable masks is not recommended because of the potential risk of wearing a SARS-CoV-2-contaminated mask. Most of these reports focused on the disinfection of masks in medical institutions [19,20,21,22]. However, reuse of disposable masks is not recommended because of the potential risk of wearing a SARS-CoV-2-contaminated mask.

### 1.3. Factors Associated with Reuse of Masks

The supply of masks to the general population during the COVID-19 pandemic has been challenging due to the shortage of mask production [23], thus resulting in the unhealthy behavior of reusing masks. However, studies that have examined the factors associated with mask reuse during the pandemic are limited.

A cross-sectional study enrolling 3981 non-healthcare workers in Brazil found that 71.1% of individuals reused masks during the COVID-19 pandemic and that women were less likely to reuse masks than men [24]. Another cross-sectional descriptive study involving 1000 adults in Hong Kong found that 35.4% of the subjects reused face masks during the COVID-19 pandemic, and individuals with inadequate face masks were more likely to reuse masks [25]. However, the previous study enrolled only non-healthcare workers and was inadequately controlled for potential confounders, such as knowledge on the usage of masks.

This study aimed to understand the factors associated with mask reuse and provide important information for educating individuals regarding appropriate preventive behaviors against the SARS-COV-2 infection. Therefore, we conducted a nationwide survey to determine the prevalence and factors associated with mask reuse in Taiwanese citizens during the COVID-19 pandemic.

## 2. Materials and Methods

### 2.1. Sampling and Data Collection

This study recruited adult Taiwanese citizens who were interviewed for COVID-19-preventive behaviors and knowledge on mask usage from 18 May through 31 May 2020. A computer-assisted telephone interview system was used to randomly select participants from 23 million Taiwanese population [26,27,28]. Based on the Cochran formula, a sample size of 1075 was adequate to attain a confidence level of 95% and a margin of error of 5% for a population of 19,338,629 Taiwan adults aged ≥20 years. The distribution of participants across regions of Taiwan in this study was similar to that of the entire Taiwanese population in 2020 (*p* = 0.78; Table A1). This study was approved by the Institutional Review Board of Taipei City Hospital (no. TCHIRB-10904012-E), and all methods were performed in accordance with TCH-IRB guidelines and regulations.

### 2.2. Preventive Behaviors against SARS-CoV-2 Infection

This study evaluated participants’ preventive behaviors against SARS-CoV-2 infection by using five items: wearing masks (yes/no), reducing trips to public places (yes/no), disinfecting hands with alcohol-based hand sanitizer (yes/no), improving personal immunity (e.g., getting enough sleep) (yes/no), and purchasing masks (yes/no). Each item scored one point if the participant practiced it. The preventive behavior scores against SARS-CoV-2 infection in study participants were summed and ranged from 0 to 5. Higher scores indicated the better practice of preventive behaviors against SARS-CoV-2 infection among the study participants.

### 2.3. Data Collection

At the time of enrollment for the study, participants underwent a computer-assisted telephone interview conducted by a trained case manager using a standardized questionnaire. This questionnaire was used to collect information about the participants’ sociodemographic characteristics, preventive behaviors against SARS-CoV-2 infection, the number of masks demanded per week, knowledge on mask usage, and the reuse of masks before and during the COVID-19 pandemic. The sociodemographic characteristics included age, sex, region of residence, employment, marital status, and income. Region of residence included north, center, south, and east of Taiwan. The categories for marital status were unmarried, married, divorced, and widowed. The income level was classified as low (<10,000 New Taiwan Dollars (NTD)), intermediate (10,000 NTD to <50,000 NTD), and high (≥50,000 NTD). The number of masks demanded per week was determined by asking participants, “how many masks do you need per week to feel secure for the prevention of COVID-19 infection?”

The participants’ knowledge on mask usage was evaluated using five mask-related questions (Table A2). Participants were asked if they agreed that “it is important to wash hands before and after wearing a mask.” Moreover, the participants were asked whether they agreed that “masks can be reused after disinfection with 75% ethanol [29].” Each question scored one point if it was answered correctly by the participant. The knowledge scores on mask usage among the study participants were summed, and the total ranged from 0 to 5. Higher scores indicated better knowledge on mask usage.

### 2.4. Outcome Variables

The primary outcome of this study was the reuse of masks, which was defined as using the same mask more than two times on different occasions [24].

### 2.5. Statistical Analyses

Descriptive statistics were used to describe the study participants’ demographics, knowledge on mask usage, and mask reuse. Continuous data were presented as mean (standard deviation (SD)), and categorical variables were expressed as percentages. Multivariate logistic regression was used to determine the factors associated with mask reuse after adjusting for the participants’ age, sex, income, and marital status. Since the generalized estimating equations (GEE) method can determine the relationship between independent variables and dependent variables at various time points, our study used GEE to analyze mask reuse before and during the COVID-19 pandemic among the study participants. An adjusted odds ratio (AOR) is an odds ratio that controls other independent variables in a regression model for binary outcomes. Adjusted odds ratios (AORs) with 95% confidence intervals (CIs) were reported to show the strength and direction of these associations. The *p*-value is the marginal significance level of statistical hypothesis testing and represents the probability of an occurring event. The variable with *p* < 0.05 was defined as a significant factor associated with mask reuse in the multivariate analysis. All data management and analyses were performed using the SAS 9.4 statistical software package (SAS Institute, Cary, NC, USA).

## 3. Results

### 3.1. Participant Selection and Characteristics

This study included 1075 individuals who were interviewed for COVID-19 preventive behaviors and knowledge on mask usage from 18 May through 31 May 2020. The mean (SD) age was 57.36 (15.61) years, and 63.2% of the participants were female. The mean (SD) number of masks demanded per week was 4.97 (2.47) (Table 1). Most (82.2%) participants reported mask reuse during the pandemic, which was significantly higher than that before the pandemic (72.7%; *p* < 0.001).

The most frequently practiced behavior against SARS-CoV-2 infection among the study participants was mask usage (72.8%), followed by reducing trips to public places (49.0%) and disinfecting hands with an alcohol-based hand sanitizer (47.7%). The mean (SD) preventive behavior score against SARS-CoV-2 infection was 2.26 (1.79).

### 3.2. Knowledge on Mask Usage during the COVID-19 Pandemic

**Figure 1:**
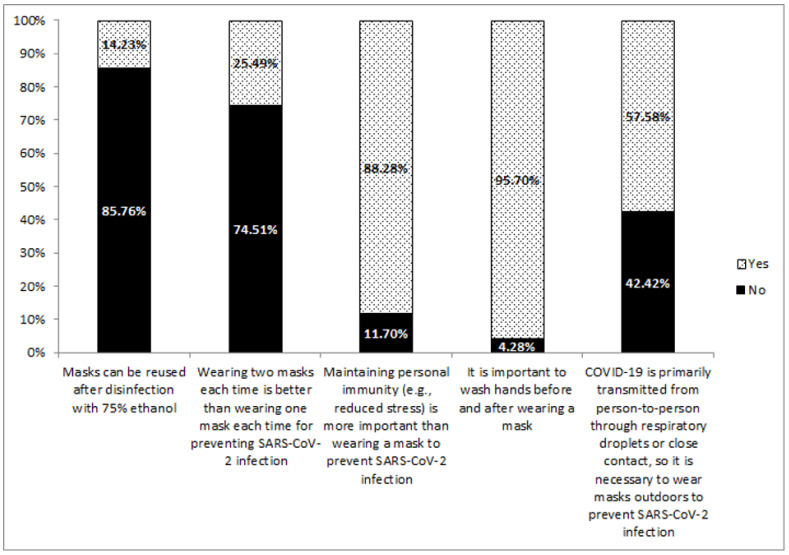
Knowledge on mask usage among study participants during the COVID-19 pandemic in Taiwan (*N* = 1075).

The mean (SD) knowledge score on mask usage was 3.10 (0.94). Most (95.7%) participants agreed that it is important to wash hands before and after wearing masks (Figure 1). Moreover, 85.8% of study participants disagreed that ethanol can decontaminate masks for reuse.

### 3.3. Factors Associated with the Reuse of Masks during the COVID-19 Pandemic

Multivariate logistic regression was used to identify the independent factors associated with the reuse of masks in study participants. After controlling the demographic characteristics and other covariates, participants were less likely to reuse masks if they had great knowledge on mask usage (AOR = 0.83, 95% CI = 0.70‒0.99; *p* = 0.04) (Table 2). The higher the knowledge score on masks usage, the less likely the reuse of face masks. Moreover, individuals with a high supply of masks were less likely to reuse masks during the COVID-19 pandemic (AOR = 0.82, 95% CI = 0.77‒0.87; *p* < 0.001). The higher the number of mask demands per week, the lower the probability of face mask reuse. Other independent predictors for mask reuse included female sex (AOR = 1.57; 95% CI = 1.11‒2.22).

### 3.4. Reuse of Masks before and during the COVID-19 Pandemic

Table 3 shows the generalized estimating equation model for the analysis of mask use before and during the pandemic. After controlling the demographic characteristics and other covariates, individuals were more likely to reuse masks (AOR = 1.75; 95% CI = 1.37‒1.75) during the COVID-19 pandemic than before the pandemic.

## 4. Discussion

Our study found that 82.2% of individuals reused masks during the COVID-19 pandemic in Taiwan. Compared with their mask-wearing behavior before the COVID-19 pandemic, people were 1.7-fold more likely to reuse masks during the COVID-19 pandemic. Moreover, individuals who had greater knowledge on mask usage or had a high supply of masks were less likely to reuse masks during the COVID-19 pandemic.

The prevalence of mask reuse among the Taiwanese citizens was 82.2% during the COVID-19 pandemic, which was higher than the prevalence reported (71.1%) among Brazilian citizens [24] and among Hong Kong citizens (34.5%) [25]. Although wearing masks is an important strategy to control the spread of SARS-CoV-2 before an adequate supply of vaccines [15], it is not recommended that disposable masks be reused due to the potential risk of wearing SARS-CoV-2-contaminated masks. A recent report showed that SARS-CoV-2 can survive on masks for as long as seven days [18]. Since reusing masks is highly prevalent during the COVID-19 pandemic, the findings of our study suggest that it is imperative to educate people about proper behaviors related to mask usage to prevent SARS-CoV-2 infection.

Our study found that individuals were less likely to reuse masks if they had a good knowledge of mask usage. In total, 85.8% of participants disagreed that masks can be reused after disinfection with 75% ethanol. Wearing masks is the key component of NPI to prevent and control the COVID-19 outbreak [30]. Correct knowledge on mask usage not only protects individuals from being infected with SARS-COV-2 but also limits the spread of COVID-19 in the community. Although ethanol 70–95% had an efficient viricidal activity against SARS-COV-2 [31], mask decontamination using ethanol can decrease the filter efficiency and negatively affect the protective effect of masks [29]. Concerning emerging infectious diseases, it is extremely important for the public to have proper awareness and employ self-protection behaviors for community prevention [32].

The results of this study showed that individuals had a lower likelihood of reusing masks when they had a high supply of masks. During the COVID-19 pandemic, there has been a shortage of masks in many countries [23], including Taiwan [33]. The insufficient supply of masks to the general population can lead to mask reuse. Because mask reuse is associated with a higher risk of acquiring COVID-19, the government should take appropriate measures to increase the production of masks and the availability to the general population to reduce the reuse rate. A recent report in Hong Kong suggests that a stock of 90 face masks per person is adequate to avoid the reuse of masks during the COVID-19 pandemic [25].

This study showed that people were more likely to reuse masks during the COVID-19 pandemic than they did before the pandemic. Wearing masks is the mainstay NPI to help prevent COVID-19 infection [30]. Different countries have different attitudes and strategies regarding mask usage against SARS-COV-2 infection [16]. In the USA, wearing masks is recommended for riding on public transportation, health care workers, older adults or people with certain underlying medical conditions, and working at a job with large numbers of the public [34]. Moreover, an N95 respirator is recommended for healthcare personnel who need protection from SARS-COV-2 infection. In areas where the virus is circulating, disposable mask or fabric masks should be worn in crowded settings [34]. The Taiwan government has recommended wearing face masks among the healthy general population and mandated masks in public places, such as healthcare facilities and markets [7,35]. However, a high proportion of individuals have been reusing face masks during the COVID-19 pandemic.

Some limitations should be considered when interpreting the findings of this nationwide study. First, the cross-sectional study design prevented us from determining the causality between mask reuse and associated factors. Second, this study determined the prevalence and factors associated with mask reuse in adult citizens before the COVID-19 vaccine was available. Future research is needed to evaluate COVID-19-preventive behaviors and knowledge on mask usage in individuals after the availability of the COVID-19 vaccine. Finally, the external validity of our findings may be a concern, as all our subjects were Taiwanese. The generalizability of our results to other non-Asian ethnic groups requires further verification. However, our findings suggest new avenues for future research.

## 5. Conclusions

This study found that 82.2% of individuals reused masks during the COVID-19 pandemic in Taiwan. Reusing a face mask is related to an insufficient supply of masks and inadequate knowledge on mask usage. Future research is necessary to determine the quantity of masks that should be supplied to each individual avoid the unhealthy behavior of reusing masks. Moreover, the implementation of educational programs from the government is imperative to educate people on the correct knowledge and proper behavior of using masks.

## Figures and Tables

**Table 1:** General characteristics of study participants in a nationwide survey for COVID-19-preventive behavior and knowledge on usage of masks in Taiwan (*N* = 1075).

Characteristics	n (%) *
Demographics	
Age, years	
Mean ± SD	57.36 ± 15.61
20–39	170 (15.80)
40–49	148 (13.80)
50–59	216 (20.10)
≥60	541 (50.30)
Sex	
Male	396 (36.80)
Female	679 (63.20)
Region of residence in Taiwan	
North	483 (44.90)
Center	211 (19.60)
South	316 (29.40)
East	65 (6.00)
Employment	
No	575 (53.50)
Yes	500 (46.50)
Marital status	
Unmarried	173 (16.10)
Married	836 (77.80)
Divorced/Widowed	66 (6.10)
Income level ^a^	
Low (<10,000 NTD)	363 (33.80)
Intermediate (10,000 NTD to <50,000 NTD)	570 (53.02)
High (≥50,000 NTD)	142 (13.20)
Number of masks demanded per week (Mean ± SD)	4.97 ± 2.47
Practicing behaviors against SARS-CoV-2 infection	
Wearing masks	783 (72.80)
Reducing trips to public places	527 (49.00)
Disinfecting hands with alcohol-based hand sanitizer	513 (47.70)
Improving personal immunity (e.g., getting enough sleep)	317 (29.50)
Purchasing masks	289 (26.90)
Preventive behaviors score against SARS-CoV-2 infection (Mean ± SD)	2.26 ± 1.79
Outcomes	
Reuse of mask before COVID-19 pandemic	781 (72.65)
Reuse of mask during COVID-19 pandemic	884 (82.23)

COVID-19, coronavirus disease; SD, standard deviation; SARS-CoV-2, severe acute respiratory syndrome coronavirus 2; * Unless stated otherwise. ^a^ The income level is presented in New Taiwan Dollars (NTD) dollars. (US dollars:NT dollars = 1:27.85).

**Table 2:** Univariate and multivariate analyses of factors associated with the reuse of masks during the COVID-19 pandemic in Taiwan (*N* = 1075).

Variables	Number of Subjects	Reuse of Mask	Univariate Analysis	Multivariate Analysis
		n (%)	OR (95% CI)	AOR (95% CI)
Knowledge score on usage of masks (per score increase)	–	–	0.87 (0.74–1.03)	0.83 (0.70–0.99) *
Preventive behaviors score against SARS-CoV-2 infection (per score increase)	–	–	0.96 (0.88–1.05)	0.96 (0.88–1.05)
Number of masks demanded per week (per number increase)	–	–	0.84 (0.79–0.89) ***	0.82 (0.77–0.87) ***
Age, years				
20–39	170	139 (81.76)	1.00 (ref)	1.00 (ref)
40–49	148	127 (85.81)	1.35 (0.74–2.47)	1.27 (0.66–2.44)
50–59	216	182 (84.26)	1.19 (0.70–2.04)	1.11 (0.61–2.03)
≥60	541	436 (80.59)	0.93 (0.59–1.44)	0.89 (0.51–1.56)
Sex				
Male	396	314 (79.30)	1.00 (ref)	1.00 (ref)
Female	679	570 (84.00)	1.37 (0.99–1.88)	1.57 (1.11–2.22) *
Region of residence in Taiwan				
North	483	402 (83.23)	1.00 (ref)	1.00 (ref)
Center	211	176 (83.41)	1.01 (0.66–1.56)	0.93 (0.59–1.45)
South	316	258 (81.65)	0.90 (0.62–1.30)	0.91 (0.62–1.33)
East	65	48 (73.85)	0.60 (0.31–1.04)	0.53 (0.28–0.99) *
Employment				
No	575	465 (80.87)	1.00 (ref)	1.00 (ref)
Yes	500	419 (83.80)	1.22 (0.89–1.68)	1.21 (0.78–1.89)
Marital status				
Unmarried	173	143 (82.66)	1.00 (ref)	1.00 (ref)
Married	836	688 (82.30)	0.98 (0.63–1.50)	1.09 (0.65–1.81)
Divorced/Widowed	66	53 (80.30)	0.86 (0.42–1.76)	0.85 (0.38–1.90)
Income level ^a^				
Low (<10,000 NTD)	363	288 (79.34)	1.00 (ref)	1.00 (ref)
Intermediate (10,000 NTD to <50,000 NTD)	570	483 (84.74)	1.45 (1.03–2.03) *	1.45 (0.96–2.20)
High (≥50,000 NTD)	142	113 (79.58)	1.02 (0.63–1.64)	0.91 (0.50–1.64)

COVID-19, coronavirus disease; SD, standard deviation; SARS-CoV-2, severe acute respiratory syndrome coronavirus 2; OR, odds ratio; CI, confidence interval. ^a^ The income level is presented in New Taiwan Dollars (NTD) dollars. (US dollars:NT dollars = 1:27.85). * *p* < 0.05; *** *p* < 0.001.

**Table 3:** Generalized estimating equation (GEE) model for the analysis of reuse of masks before and during the COVID-19 pandemic in Taiwan (*N* = 1075).

Variables	AOR (95% CI)
Period of reuse of masks	
Before COVID-19 pandemic	1.00 (ref)
During COVID-19 pandemic	1.75 (1.37‒1.75) ***
Age, years	
20–39	1.00 (ref)
40–49	1.10 (0.74‒1.57)
50–59	0.97 (0.70‒1.36)
≥ 60	1.03 (0.75‒1.41)
Sex	
Male	1.00 (ref)
Female	1.32 (1.01‒1.52) *
Region of residence in Taiwan	
North	1.00 (ref)
Center	1.06 (0.81‒1.35)
South	0.95 (0.76‒1.20)
East	0.72 (0.52‒1.16)
Employment	
No	1.00 (ref)
Yes	1.10 (0.84‒1.38)
Marital status	
Unmarried	1.00 (ref)
Married	1.26 (0.91‒1.58)
Divorced/Widowed	1.59 (0.87‒2.35)
Income level ^a^	
Low (<10,000 NTD)	1.00 (ref)
Intermediate (10,000 NTD to <50,000 NTD)	1.26 (0.91‒1.58)
High (≥50,000 NTD)	1.59 (0.87‒2.35)

COVID-19, coronavirus disease; AOR, adjusted odds ratio; CI, confidence interval. ^a^ The income level is presented in New Taiwan Dollars (NTD) dollars. (US dollars:NT dollars = 1:27.85. * *p* < 0.05; *** *p* < 0.001.

## Data Availability

The data for the study are available by contacting the corresponding author upon request.

## Appendix A

**Table A1:** Comparison of the distribution of residence between study participants and the general population of Taiwan.

Region	Taiwan Population		Study Sample	
	*N*	%	*N*	%
North	8,826,374	45.64	483	44.93
Center	3,704,649	19.16	211	19.63
South	5,761,657	29.79	316	29.40
East	1,045,949	5.41	65	6.05
Total	19,338,629	100.00	1075	100.00

**Table A2:** Evaluation of knowledge on mask usage during the COVID-19 pandemic.

Questions	Score
COVID-19 is primarily transmitted from person to person through respiratory droplets or close contact, so it is necessary to wear masks outdoors to prevent SARS-CoV-2 infection ^a^	
No	1
Yes	0
It is important to wash hands before and after wearing a mask.	
No	0
Yes	1
Maintaining personal immunity (e.g., reduced stress) is more important than wearing a mask to prevent SARS-CoV-2 infection	
No	1
Yes	0
Wearing two masks each time is better than wearing one mask each time for preventing SARS-CoV-2 infection ^b^	
No	1
Yes	0
Masks can be reused after disinfection with 75% ethanol ^c^	
No	1
Yes	0

COVID-19, coronavirus disease; SD, SARS-CoV-2, severe acute respiratory syndrome coronavirus 2. ^a^ Centers for Disease Control and Prevention (CDC). Guidance for Wearing Masks, available from: https://www.cdc.gov/coronavirus/2019-ncov/prevent-getting-sick/cloth-face-cover-guidance.html (accessed on 12 March 2021). ^b^ Centers for Disease Control and Prevention (CDC). Improve How Your Mask Protects You: Available from: https://www.cdc.gov/coronavirus/2019-ncov/your-health/effective-masks.html (accessed on 30 March 2021). ^c^ Grinshpun et al. Autoclave sterilization and ethanol treatment of re-used surgical masks and N95 respirators during COVID-19: impact on their performance and integrity. The Journal of hospital infection. 2020;105(4):608-614.
